# P-1874. Impact of Compromised Host AntimicRobial Management Service (CHARMS) on All Cause Readmission in Solid Organ Transplant Patients Discharged on Intravenous Antimicrobials

**DOI:** 10.1093/ofid/ofae631.2035

**Published:** 2025-01-29

**Authors:** Brent W Footer, Luther A Bartelt, Anne Friedland, Anne Lachiewicz, Arlene C Seña, Tessa Andermann, Elizabeth C Arant, Mary Catherine Bowman, Zack Deyo, David van Duin

**Affiliations:** University of North Carolina Medical Center, Chapel Hill, North Carolina; University of North Carolina School of Medicine, Chapel Hill, NC; UNC School of Medicine, Chapel Hill, North Carolina; University of North Carolina, Chapel Hill, NC; Division of Infectious Diseases, Department of Medicine, University of North Carolina at Chapel Hill School of Medicine, Chapel Hill, NC; University of North Carolina, Chapel Hill, NC; University of North Carolina at Chapel Hill, Chapel Hill, NC; UNC, Chapel Hill, North Carolina; University of North Carolina Medical Center, Chapel Hill, North Carolina; University of North Carolina at Chapel Hill, Chapel Hill, NC

## Abstract

**Background:**

The Compromised Host AntimicRobial Monitoring Service (CHARMS) was created to support safe, efficient, streamlined care for immunocompromised patients discharged on outpatient parenteral antimicrobial therapy (OPAT). However, outcome data for OPAT programs in solid organ transplant (SOT) is lacking and to our knowledge no published literature specific to this patient population exists. The purpose of this study is to evaluate the effect of CHARMS on hospital readmission in SOT patients discharged on OPAT.

**Table 1.**
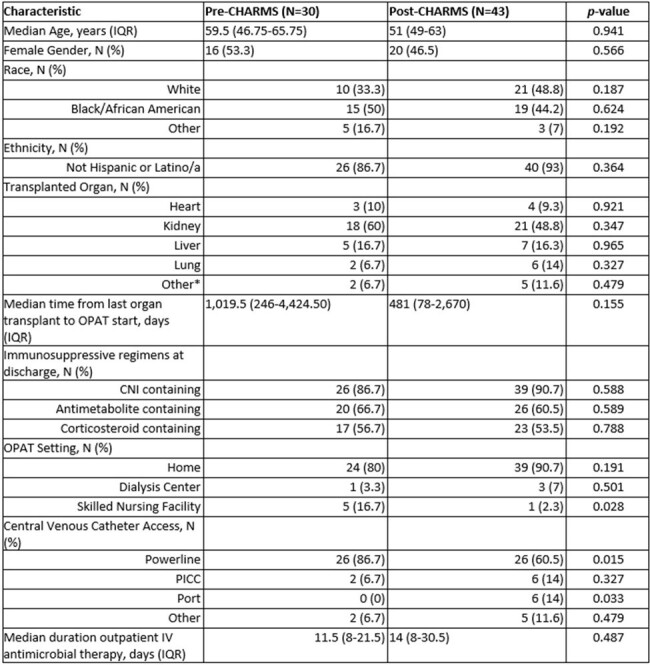
Baseline Characteristics at Start of OPAT Course *Includes patients with multiple organs and/or multiple transplants of the same organ

**Methods:**

This study utilized a quasi-experimental study design with pre- and post-CHARMS implementation periods of 1/1/23-6/30/2023 and 9/1/23-4/30/24, respectively. No patients were enrolled during the two-month period of CHARMS implementation. All adult SOT patients seen in consultation by infectious diseases and discharged from UNC Medical Center on IV antimicrobials were included. The primary endpoint was 30-day all cause hospital readmission. Secondary endpoints were all cause hospital readmission over the entirety of the OPAT course and central venous catheter (CVC) complications.

**Table 2.**
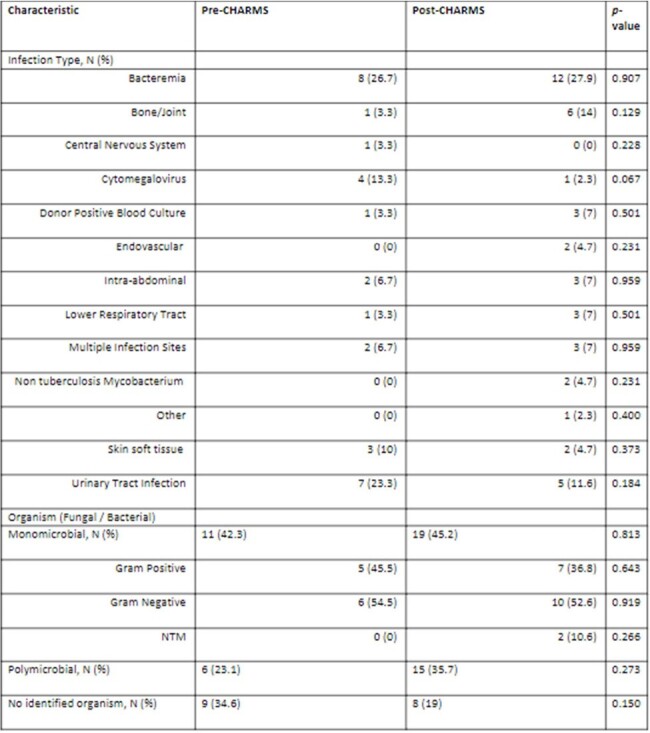
Baseline Infection Related Characteristics

**Results:**

30 and 43 OPAT courses were included in the pre- and post-CHARMS cohorts, respectively. There were minimal differences in baseline characteristics with no difference in the median duration of IV antimicrobials (Table 1). Approximately 25% of patients in each group were treated for bacteremia and more patients in the pre-intervention group were treated for cytomegalovirus (13.3% vs. 2.3%, p=0.067) (Table 2). More patients in the post-intervention group received >1 IV antimicrobial (39.5% vs. 20%, p=0.036) (Table 3). In terms of outcomes, 30-day all cause hospital readmission was decreased in the post-intervention group (pre: 36.7% vs. post: 25.6%, p=0.310). Similarly, a decrease in all cause hospital readmission over the entirety of the OPAT course was observed in the post-intervention group (pre: 43.3% vs. post: 25.6%, p=0.112). CVC complications did not differ between the groups (pre: 10% vs. post: 11.6%, p=0.827).

**Table 3.**
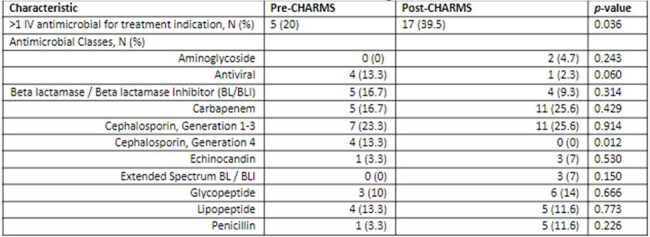
Antimicrobials Received During OPAT

**Conclusion:**

There was trend towards decreased 30-day all cause readmission in SOT patients managed by a dedicated compromised host OPAT service. Additional patients are needed to evaluate the full impact of the program.

**Disclosures:**

David van Duin, MD, PhD, Merck: Advisor/Consultant|Merck: Grant/Research Support|Pfizer: Advisor/Consultant|Qpex: Advisor/Consultant|Roche: Advisor/Consultant|Shionogi: Advisor/Consultant|Shionogi: Grant/Research Support

